# Dorsal oral mucosa graft urethroplasty for female urethral stricture reconstruction: A narrative review

**DOI:** 10.3389/fsurg.2023.1146429

**Published:** 2023-03-21

**Authors:** Chunqin Tao, Xiaoxiang Jin, Hengshu Zhang

**Affiliations:** ^1^ Department of Burns and Plastic Surgery, The First Affiliated Hospital of Chongqing Medical University, Chongqing, China; ^2^ Department of Urology, The First Affiliated Hospital of Chongqing Medical University, Chongqing, China

**Keywords:** female urethra stricture, oral mucosa graft, dorsal urethroplasty, urethral reconstruction, narrative review

## Abstract

Female urethral stricture is currently a challenging situation. In general, urethra dilatation can be selected for treatment, but the complications and high recurrence rate urge doctors to consider other treatments. Recently, dorsal oral mucosa graft urethroplasty is concerned by more and more surgeons, but there are not enough reports so far. A comprehensive search of dorsal oral mucosa graft urethroplasty was performed. According to the existing literature, there are applications of buccal mucosa and lingual mucosa, and compared with other kinds of grafts, the success rate is higher. However, there is a lack of multicenter, large sample and long follow-up studies. And there is still no enough comparative study between different types of oral mucosa. In summary, dorsal oral mucosa graft urethroplasty is an effective option for the management of female urethral stricture. More multicenter and large sample studies with long-term follow-up data are needed.

## Introduction

Female urethral stricture (FUS) is a rare and challenging problem in reconstructive surgery or urology. FUS accounts for 4% and 13% of the causes of bladder outlet obstruction (BOO) (1–6). The real prevalence of FUS is unknown, but it is normally considered a rare disease, and the absence of large series makes FUS underestimated (1, 7). Osman et al. proposed a descriptive definition of urethral stricture as “a symptomatic anatomical narrowing of the urethra based on failure in catheterization, urethral calibration, endoscopic or radiographic vision” (1).

The traditional treatment is urethra dilatation, but it may cause damage, and the cure rate is less than 50% (1, 3). Therefore, urethroplasty has gradually become a popular treatment in recent years (3, 8), There are a variety of surgical methods, including ventral, dorsal, mini-dorsal and dorsolateral approaches, etc. The types of grafts are also varied, such as vaginal mucosa reported by Tsivian et al., and oral mucosa reported by many studies (9–12). In the existing literature on dorsal oral mucosa graft (DOMG) urethroplasty, there are few serious postoperative complications, including restenosis and urinary incontinence. Despite the small sample size and the short follow-up, it seems that the use of oral mucosa is effective in this kind of operation (1, 3). This article intends to review the application of DOMG urethroplasty and summarize the existing literatures.

## Etiology

The potential etiologies of female urethral stricture disease are idiopathic, iatrogenic, traumatic, inflammatory, or malignant. In a systematic review of FUS, etiology was defined in a total of 271 patients out of 554 as idiopathic in 139 (51.3%), iatrogenic in 89 (32.8%), inflammatory/infective in 25 (9.2%), and traumatic in 18 (6.6%) (13). Iatrogenic injuries include anterior vaginal or urinary tract surgery, urinary catheterization and radiotherapy of pelvic malignant tumors (14). Traumatic urethral injuries often result from obstetric complications. Infection or inflammation can cause peri-urethral fibrosis and lead to stricture (15). For the women with vaginal mass or suspicious urothelial changes, the malignancy should be considered as a cause of urethral stricture (15).

## Methods

For this review article, a literature search was conducted in January 2023, using the Pubmed and EMBASE databases. the searching terms were “urethral stricture” AND “Female” AND [“oral mucosa” OR “buccal mucosa” OR “lingual mucosa” OR “labial mucosa”] AND “dorsal urethroplasty”. Studies were limited to humans, women, and the language must be English. Studies were reviewed independently by the authors.

## Surgical technique

### Dorsal oral mucosa graft urethroplasty

Gomez et al. and Kore et al. reported their detailed description of the technique (3, 16). The patient was under general anesthesia and in lithotomy position. Then methylene blue was injected for a better visualization of the mucosa. After placing a hydrophilic guidewire, an inverted *U*-shaped incision was made between the urethra and the clitoris, from 9 o'clock to 3 o'clock. Dissection was performed between the inferior margin of the pubis and the dorsal tissue around the urethra, until the exposure of the bladder neck. Then, at 12 o'clock of the urethra, the dorsal side of the urethra was longitudinally cut from the midline until the site of stricture with the help of catheter. In this process, the surgeon should be careful to protect the urethral tube, the clitoris and the sphincter muscle. Gomez et al. emphasized that if there was no stricture around the meatus, the incision could begin 1 cm proximal to preserve the original structure (3). Then the oral mucosal side was placed towards the urethral lumen. The edges of the graft were sutured to the urethra, including the mucosa and lateral periurethral tissues. Additionally, the graft could be quilted to the undersurface of the clitoris.

### The harvest of oral mucosa

Different types of oral mucosa are harvested in different ways. Generally, the buccal mucosa is harvest from the inner cheek and the size of the graft is determined by the stricture length (3, 17). Before the use, the graft needs to be defatted. As for the harvest of lingual mucosa, Sharma et al. reported their experience (11). After the oro-tracheal intubation, the tongue was pulled out of the mouth. The required lingual mucosa graft was harvested using a right-angled scissor. Like buccal mucosa, all the submucosal adventitial tissues need to be removed before the use.

## Current literatures

Most authors do not have clear criteria to define successful outcomes, so they resort to no further intervention and symptom relief. Patients with normal urinary flow pattern, maximum urinary flow rate > 12∼15 ml/s, urinary tract diameter > 17F, or normal anatomical imaging data are considered successful (13). As shown in Table 1, in 2006, Tsivian et al. first reported their experience in dorsal buccal mucosa graft (DBMG) urethroplasty (12). The patient was a 60-year-old woman with a history of recurrent urinary tract infection and obstructive voiding symptoms. The surgical team harvested the buccal mucosa which was 1.5 cm in width and 3.5 cm in length. Then the DBMG urethroplasty was performed. After 3 weeks, the suprapubic catheter was removed after voiding cystourethrogrphy (VCUG) showing a patent urethra. No additional treatment was required after 27-month follow-up. Migliari et al. also reported three cases of DBMG urethroplasty (9). The success rate was 100% after 6-month follow-up.

**Table 1:** Current literature of dorsal oral mucosa graft urethroplasty.

Author	Year	Patients/n	Age/years (range)	Follow-up/months (range)	Graft	Technique	Success	Complications (*n*)
Migliari et al. (9)	2006	3	53.7 (45–65)	6	BMG	Dorsal	100	None
Tsivian et al. (12)	2006	1	60	27	BMG	Dorsal	100	None
Sharma et al. (11)	2009	15	42 (25–65)	12	LMG	Dorsal	93	Wound infection (1)
Onol et al. (18)	2011	2	\	30 (24–36)	BMG	Dorsal	100	None
Blaivas et al. (19)	2012	3	\	25	BMG	Dorsal	100	None
Goel et al. (20)	2013	8	40.6	14.8 (3–24)	BMG	Dorsal	62.5	None
Kowalik et al. (21)	2014	4	51 (32–74)	34	BMG	Dorsal	100	None
Hampson et al. (17)	2019	39	50 (29–81)	33 (7–106)	BMG	Dorsal	77	Urinary tract infection (7)
Gomez et al. (3)	2019	15	51 (32–76)	15 (2–149)	BMG	Dorsal	87	Intraoperative bleeding (1)
Berdondini et al. (22)	2021	13	56 (29–69)	11 (7–18)	BMG	Mini-Dorsal	100	None
Richard et al. (10)	2021	19	\	12 (1–49)	BMG/LMG	Dorsal	89.5	Stress urinary incontinence (1) Sexual dysfunction (1)
Gupta et al. (23)	2021	3	\	12	BMG	Dorsal	100	Transient stress incontinence (1)
Kore et al. (16)	2022	21	45	25	BMG	Dorsal	90.5	Urinary tract infection (2)

BMG, buccal mucosa graft, LMG, lingual mucosa graft.

In 2009, Sharma et al. first reported their experience in dorsal lingual mucosa graft (DLMG) urethroplasty (11). 15 female patients who had undergone multiple urethral dilatations were selected for DLMG urethroplasty. The mean length of harvested mucosa was 2.95 cm. After about 15 days, all the patients underwent a trail of VCUG to establish a urethra. The success rate was 93% after 3-month follow-up with one patient having submeatal stenosis who required urethral dilatation. At the 1-year follow-up, none of the patients had any complications.

In recent ten years, the DOMG urethroplasty had become an effective and low-morbidity option for FUS. A few of literatures reported their cases of dorsal urethroplasty with buccal or lingual mucosa. All of them showed high success rates and low complication rates (3, 10, 16, 22–18). In 2019, Hampson et al. reported 39 cases of DBMG urethroplasty from multiple centers, which is the largest series for this technique (17). The mean stricture length was 2.1 cm. After mean duration of 17 days, the catheter was removed. The mean postoperative follow-up was 33 months. Stricture recurrence was seen in 9 patients with mean time to recurrence 14 months. The whole success rate was 77% without serious postoperative complications.

## Discussion

### Advantages

FUS is a rare disease. Traditionally, the treatment is symptomatic treatment, such as urethra dilatation. However, this method cannot achieve the effect of radical cure. The recurrence rate is more than 50%, and it may cause some trauma to the urethra and surrounding tissue, aggravating the formation of fibers (1, 8, 24, 25). On the contrary, urethroplasty have a high success rate in the existing reports(1, 3). Therefore, surgery should be the first choice for recurrent FUS (8).

For female urethroplasty, the option grafts include vaginal grafts, vaginal flaps or oral mucosa. Vaginal or labial flap was the most common urethroplasty technique. The advantages of local flaps are that they are mobile, usually well vascularized, and can be raised with minimal morbidity. Studies have demonstrated this technique could reach near complete resolution of symptoms (15, 18, 26–28). When vaginal fibrosis or atrophy precludes the use of a local flap, oral mucosa could be great replacement and is getting more and more popular in recent years. Oral mucosa is a natural mucosal tissue, which is elastic, hairless, easy to obtain and large enough. What's more, the process of harvesting has little damage to the donor area. That's why it is an ideal graft for urinary reconstruction (3). In recent years, oral mucosa has also been widely used in the reconstruction of male urethral stricture and the results are optimistic (29, 30). Oral mucosa is also used in the reconstruction of ureteral stricture (31). Recently, Liang et al. reported their results of 41 cases of ureteroplasty with lingual mucosa, which proved the universality and effectiveness of oral mucosa (32). As all of these materials gives good results in the urethral stricture treatment, the selection of specific materials is determined according to the specific clinical conditions and the wishes of patients.

The choice of approach is also very important. In fact, technically speaking, ventrally or dorsally is feasible (33). But recent studies have shown that dorsal approach may have more advantages: 1. The dorsal approach can avoid the occurrence of urethrovaginal fistula; 2. the graft has a firm support from the flat ventral surface of the clitoris; 3. The blood supply of the dorsal approach is more abundant, which is beneficial to the survival of the graft; 4. If patients with stress urinary incontinence need to undergo sling surgery, the ventral approach may make it difficult to perform sling surgery while the dorsal approach can avoid this problem (9, 11, 12, 20, 25, 34). Therefore, DOMG urethroplasty is accepted by more and more surgeons and widely used in clinic.

### Limitations

The current limitations are obvious. The number of cases in the existing literature is small, and most of them are single-center studies, and the follow-up time is not long enough. There is a lack of high-quality studies with multi-center, large sample size and long follow-up time.

In addition, there are many types of oral mucosa, including labial mucosa, lingual mucosa and buccal mucosa. The survival rate of different mucosa and its effect on operation are also different. In male urethral reconstruction, it has been reported that the overall result of lingual mucosa is similar to buccal mucosa (35, 36). In ureteral reconstruction, several studies have demonstrated that ureteroplasty using lingual mucosa grafts yields better recipient site outcomes and fewer donor site complications than that using BMG (37, 38). In the study of FUS, only Coskun et al. compared the effects of labial and buccal mucosa (39). There is a lack of more high-quality random controlled trails to further confirm the effects of different mucosa.

### Further directions

We need large sample, multi-center and long follow-up studies to demonstrate the effect of DOMG urethroplasty. Also, the comparison of different oral mucosa types is also needed, which is of great guiding significance to the clinic. As for the improvement of surgical methods, Berdondini et al. put forward their ideas (22). Considering that traditional dorsal approach ma**y** damage the sphincter or the clitoral bodies, they use a transurethral approach without dissection of the dorsal urethra from the surrounding tissues to preserve as much urethra/periurethral tissues as possible. They presented this novel surgical technique as “mini-dorsal BMG urethroplasty”. All the patients got good results in their follow-up. In the future, we need more improvements in surgical methods to make the surgery less traumatic, recover faster and benefit more patients.

## Conclusions

DOMG urethroplasty is an effective option for the management of female urethral stricture. More multicenter and large sample studies with long-term follow-up data are needed.
